# Strategies to Improve the Antitumor Effect of γδ T Cell Immunotherapy for Clinical Application

**DOI:** 10.3390/ijms22168910

**Published:** 2021-08-18

**Authors:** Masatsugu Miyashita, Teruki Shimizu, Eishi Ashihara, Osamu Ukimura

**Affiliations:** 1 Department of Urology, Kyoto Prefectural University of Medicine, Kyoto 602-8566, Japan; shimizu1@koto.kpu-m.ac.jp (T.S.); ukimura@koto.kpu-m.ac.jp (O.U.); 2 Department of Urology, Japanese Red Cross Kyoto Daini Hospital, Kyoto 602-8026, Japan; 3 Department of Clinical and Translational Physiology, Kyoto Pharmaceutical University, Kyoto 607-8414, Japan; ash@mb.kyoto-phu.ac.jp

**Keywords:** γδ T cells, immunotherapy, tumor resistance, combination therapy, tumor microenvironment, immune checkpoint inhibitor

## Abstract

Human γδ T cells show potent cytotoxicity against various types of cancer cells in a major histocompatibility complex unrestricted manner. Phosphoantigens and nitrogen-containing bisphosphonates (N-bis) stimulate γδ T cells via interaction between the γδ T cell receptor (TCR) and butyrophilin subfamily 3 member A1 (BTN3A1) expressed on target cells. γδ T cell immunotherapy is classified as either in vivo or ex vivo according to the method of activation. Immunotherapy with activated γδ T cells is well tolerated; however, the clinical benefits are unsatisfactory. Therefore, the antitumor effects need to be increased. Administration of γδ T cells into local cavities might improve antitumor effects by increasing the effector-to-target cell ratio. Some anticancer and molecularly targeted agents increase the cytotoxicity of γδ T cells via mechanisms involving natural killer group 2 member D (NKG2D)-mediated recognition of target cells. Both the tumor microenvironment and cancer stem cells exert immunosuppressive effects via mechanisms that include inhibitory immune checkpoint molecules. Therefore, co-immunotherapy with γδ T cells plus immune checkpoint inhibitors is a strategy that may improve cytotoxicity. The use of a bispecific antibody and chimeric antigen receptor might be effective to overcome current therapeutic limitations. Such strategies should be tested in a clinical research setting.

## 1. Introduction

Cancer is one of the most serious and potentially fatal diseases in humans. According to statistical reports, there were an estimated 18.1 million new cancer cases and 9.6 million cancer-related deaths worldwide in 2018 [1]. Surgery, chemotherapy, and radiotherapy are the three pillars of antitumor therapy. Surgery and radiotherapy are curative for localized cancers; however, most cancer-related deaths are due to metastasis, which requires systemic therapy. Chemotherapy is the first-line systemic therapy against metastatic cancers; however, many cancers become resistant, which leads to treatment failure. Recently, immunotherapy, now regarded as the fourth pillar of antitumor therapy, has been used for systemic antitumor therapy.

T cell-based immunotherapy is an effective cancer treatment strategy. T cells are divided into two major subpopulations based on surface expression of αβ and γδ T cell receptors (TCRs). αβ T cells recognize peptide antigens in the context of non-self; for example, antigens expressed by cancer cells. αβ T cells are effector cells that operate within the adaptive arm of the immune system; these cells exert cytotoxicity in a major histocompatibility complex (MHC)-restricted manner. However, due to loss of MHC molecules, tumor cells are often resistant to attack by αβ T cells [2]. By contrast, γδ T cells are effectors that operate within the innate arm of the immune system; these cells act in an MHC-unrestricted manner, making them interesting mediators of cancer immunotherapy. Human γδ T cells were first identified in the mid-1980s [3,4,5]. They are abundant in the intestine and skin and play a role in defense against microbial infections in an MHC-unrestricted manner [6]. Recent studies show that γδ T cells exert potent cytotoxic effects against various types of cancer cell [7,8,9,10,11,12]. Their activation induces release of cytotoxic molecules such as perforin and granzymes. Activated γδ T cells also secrete cytokines such as interferon-γ (IFN-γ) and tumor necrosis factor-α (TNF-α). These cytotoxic molecules and cytokines induce cancer cell apoptosis. However, γδ T cells comprise only a small percentage of circulating lymphocytes and require stimulation to exert antitumor effects. In this review, we will outline the methods used to stimulate γδ T cells and improve their antitumor effects. We also discuss strategies for clinical application.

## 2. Phosphoantigens and Nitrogen-Containing Bisphosphonates Stimulate γδ T Cells

Human peripheral blood γδ T cells, which predominantly express the Vδ2 chain paired with the Vγ9 chain, are activated upon recognition of phosphoantigens (PAgs) such as (E)-4-hydroxy-3-methylbut-2-enyl pyrophosphate (HMBPP), which is synthesized in bacteria via isoprenoid biosynthesis [13], and isopentenyl pyrophosphate (IPP), which is produced in eukaryotic cells via the mevalonate pathway [14]. Activation of γδ T cells by PAgs was first reported in the 1990s [15,16]; however, it is unclear how the γδ TCR recognizes PAgs. Butyrophilin subfamily 3 member A1 (BTN3A1) molecules, which are isoforms of the BTN3A (also termed CD277) subfamily, play an indispensable role in activation of γδ T cells by PAgs [17]. BTN3A1, which is expressed ubiquitously on the surface of cells, comprises two immunoglobulin-like extracellular domains and an intracellular B30.2 domain. The precise mechanism by which γδ T cells recognize BTN3A1 is not completely clear, but several studies demonstrate that binding of PAgs directly to a positively-charged pocket in the intracellular B30.2 domain of BTN3A1 recruits the cytoskeletal adaptor protein periplakin and the GTPase RhoB, which increases membrane mobility and induces a conformational change in BTN3A1; the altered conformation is recognized by the γδ TCR [18,19]. Recent studies show that BTN2A1, which binds directly to the TCRs via germline-encoded regions of Vγ9, is also essential to BTN3A-mediated γδ T cell cytotoxicity and BTN2A1 expression at the plasma membrane of cancer cells correlated with γδ T cell cytotoxicity [20,21]. BTN2A1 interacts with BTN3A1, leading to enhance plasma membrane export, and BTN2A1/BTN3A1 interaction is enhanced by PAgs. Anti-BTN2A monoclonal antibodies (mAbs) inhibit BTN2A1 biding to the γδ TCR and modulate γδ T cell killing of cancer cells [21]. These studies demonstrate the potential of butyrophilin subfamily cooperation pathway as a therapeutic target in γδ T cell activation.

**Figure 1:**
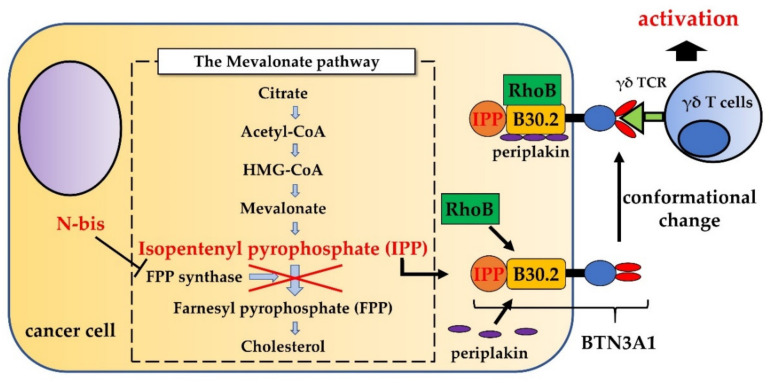
Mechanism of γδ T cell activation by N-bis. N-bis inhibits FPP synthase in the mevalonate pathway and induces accumulation of IPP. Binding of IPP to the intracellular B30.2 domain of BTN3A1 recruits the cytoskeletal adaptor protein periplakin and the GTPase RhoB, which increases membrane mobility and induces a conformational change in BTN3A1, which is then recognized by the γδ TCR.

In general, the concentrations of PAgs is not high enough to stimulate γδ T cells under physiological conditions; however, tumor cells show upregulated production of PAgs due to metabolic reprogramming, which increases mevalonate pathway activity [22,23]. Moreover, PAgs concentrations can be increased pharmacologically. Nitrogen-containing bisphosphonates (N-bis) such as pamidronate (Pam) and zoledronate acid (ZOL), which are used to treat hypercalcemia or bone metastases of cancer, inhibit the enzyme farnesyl diphosphate (FPP) synthase, which is the rate determining enzyme in the mevalonate pathway [24]. As a result, the concentration of IPP (derived from the upstream FPP synthase metabolite) increases, thereby activating γδ T cells (Figure 1).

γδ T cell-based immunotherapy is classified according to the method used to activate and expand the cells [25]. The first method involves in vivo activation by systemic administration of PAgs or N-bis, along with exogenous interleukin (IL)-2 [26,27,28,29,30,31,32] (Table 1). Dieli et al. conducted a phase I clinical trial involving patients with metastatic hormone-refractory prostate cancer. The aim was to examine the antitumor effect of single or combined administration of ZOL and IL-2. Nine patients were enrolled in each arm. Six of the nine patients received combined administration of ZOL and IL-2, but only two of nine patients received single administration of ZOL, and showed a significant long-term shift in peripheral blood γδ T cells toward an activated state in which they produced IFN-γ and perforin; also, the number of activated γδ T cells showed a significant correlation with favorable clinical outcomes [26]. This indicates the importance of the administration of IL-2 to maintain peripheral γδ T cells. Wilhelm et al. reported a pilot study of patients with low-grade non-Hodgkin lymphoma and multiple myeloma; this study involved in vivo activation of γδ T cells by combined administration of Pam and IL-2. The results showed that γδ T cell activation/proliferation and response to treatment were disappointing, with only one of ten patients that received an intravenous infusion of IL-2 on Day 3 through Day 8 achieving stable disease. On the other hand, the next nine patients selected had shown positive in vitro proliferation of γδ T cells in response to Pam/IL-2; when these patients received an intravenous infusion of IL-2 on Day 1 through Day 6, five showed in vivo activation/proliferation of γδ T cells, and three showed a partial response [27]. Therefore, if patients are to have any chance of a clinical responses, they must show positive in vitro proliferation of γδ T cells in response to stimulation with Pam, and IL-2 must be administered immediately after in vivo Pam stimulation. Lang et al. reported a pilot trial of in vivo γδ T cell activation in 12 patients with metastatic renal cell carcinoma (RCC); they used different doses of ZOL in combination with low-dose IL-2. Two patients experienced a prolonged period of stable disease; however, no objective clinical responses were observed [28]. The most common adverse events associated with in vivo-activated γδ T cell immunotherapy are the same as those reported for IL-2 monotherapy; they include fever, fatigue, elevation of liver transaminase, and eosinophilia. These adverse events are usually grade 1 or 2, meaning that in vivo-activated therapy is well tolerated. However, the clinical benefits appear to be mild to moderate [25]. This problem could be related to anergy and exhaustion of activation-induced γδ T cells. The mechanisms underlying this anergy and exhaustion remain unclear. The second category of γδ T cell immunotherapy involves ex vivo expansion of γδ T cells by PAgs or N-bis, followed by administration of the cultured γδ T cells to the patient (i.e., adoptive immunotherapy) [33,34,35,36,37,38,39,40,41,42] (Table 1). The mechanism by which N-bis expands γδ T cells from peripheral blood is as follows: treatment of peripheral blood mononuclear cells with N-bis leads to accumulation of IPP in monocytes because these cells take up N-bis efficiently; the monocytes that accumulate IPP become antigen-presenting cells and stimulate γδ T cells in the peripheral blood [43,44]. Kobayashi et al. conducted a pilot study of adoptive immunotherapy in patients with advanced RCC using autologous γδ T cells stimulated by PAg (namely, 2-methyl-3-butenyl-1-pyrophosphate (2M3B1-PP)). Seven patients were enrolled and all received an intravenous infusion of recombinant human IL-2 plus autologous γδ T cells expanded from their own peripheral blood nuclear cells. All patients had IL-2-related adverse events, which were graded as 1 or 2. The antitumor effects in five patients were evaluated by comparing the tumor-doubling time, assessed by computed tomography (CT), between pre- and post-treatment. Three of the five showed a prolonged tumor-doubling time; however, the other two patients showed a shorter tumor-doubling time. One died within 2 months of γδ T cell administration, and the other showed a shorter tumor-doubling time for liver metastases [33]. In this study, no patient received systemic ZOL. ZOL treatment is important for the antitumor effects of γδ T cells because it inhibits FPP synthase, leading to accumulation of IPP in cancer cells and specific antitumor cytolysis by γδ T cells in a TCR-dependent manner. Kobayashi et al. also conducted a phase Ι/ΙΙ study of adoptive γδ T cell immunotherapy in combination with ZOL and IL-2. Enrolled patients had advanced RCC. Eleven patients were enrolled and all received 4 mg ZOL intravenously, followed by administration of autologous γδ T cells starting 2 h after completion of ZOL infusion. Patients then received low-dose recombinant human IL-2 on Day 0 through Day 4. Clinical responses were examined by CT and evaluated using the Response Evaluation Criteria in Solid Tumors. One patient exhibited a complete response, five patients had stable disease (SD), and five had progressive disease (PD) [34]. Nicol et al. reported a clinical study of autologous γδ T cell immunotherapy for various types of metastatic solid tumors (i.e., melanoma, breast cancer, cervical cancer, ovarian cancer, colon cancer, cholangiocarcinoma, and duodenal cancer). Eighteen patients were enrolled. Three of the 14 evaluable patients showed a SD and 11 had PD. Interestingly, this study also examined the migratory pattern of intravenously-infused ex vivo-expanded γδ T cells labeled with radioactive ^111^indium oxine (^111^In) in three patients (two melanoma patients, one colon cancer patient). In all three, labeled γδ T cells migrated rapidly to the lungs and remained there for 4 to 7 h. Cell numbers (estimated by measurement of γ-ray radioactivity in the lungs) decreased slowly, corresponding with gradual migration into the liver and spleen. After 24 h, almost all cells were located in the liver and spleen and virtually no activity remained in the lungs. Moreover, assessment of the number of peripheral blood γδ T cells at multiple time points during the 48 h after γδ T cell infusion showed no substantial change compared with pre-infusion levels. These data indicate that few of the γδ T cells remained in the bloodstream. However, in one melanoma patient of the three patients, the ^111^In-labeled γδ T cells appeared to have migrated to the metastatic mass on the left adrenal gland by 1 h after infusion. Maximal activity was seen at the metastatic tumor site at 4 h, and the tracer remained detectable for 48 h [35]. Adoptive immunotherapy using ex vivo-expanded γδ T cells is also safe and well tolerated; however, expanding γδ T cells from some cancer patients is difficult. The reasons for this are unclear. Moreover, favorable clinical outcomes require higher effector (γδ T cells)-to-target cell (cancer cells) ratios (E/T ratio) at the tumor site. Although potent cytotoxic activity against various cancer cells has been confirmed in vitro, there is much room for improvement.

## 3. Administration of γδ T Cells into a Local Cavity Improves the E/T Ratio to Achieve a Maximum Cytotoxic Effect

The E/T ratio at the tumor site is an important factor that determines cytotoxicity. Administration of effector cells into a local cavity might improve the E/T ratio at the tumor site, making it more likely that γδ T cells make direct contact with cancer cells. Several studies describe administration of γδ T cells into a local cavity, such as the intraperitoneal cavity, enucleated cavity, or intravesical cavity. Wada et al. reported injection of ex vivo-expanded γδ T cells following ZOL administration into the intraperitoneal cavity of seven patients with symptomatic malignant ascites secondary to gastric adenocarcinoma. Two of the seven dropped out of the study after a single injection due to disease progression. In one patient, the bloody ascites became clear and reduced in volume. In another patient, the ascites almost disappeared. The most commonly observed treatment-related adverse events were fever and ZOL-induced hypocalcemia. These events were reversible, and none of the patients experienced abdominal pain or any toxicity related to the intraperitoneal injection of γδ T cells [38]. Nichole et al. reported intracranial infusion of ex vivo-expanded γδ T cells from healthy volunteers into athymic nude mice bearing xenografts of the human glioblastoma (GBM) cell line, U251. Intracranial infusion of γδ T cells led to regression of GBM tumors and improved survival [45]. Intravesical administration of drugs (mitomycin C, adriamycin, or Bacillus Calmette-Guerin) is the standard treatment for bladder cancers. Yuasa et al. implanted a human bladder cancer cell line (UMUC3 cells transfected with the luciferase gene (UMUC3-luc)) into the murine bladder cavity and then administered ex vivo-expanded γδ T cells from healthy volunteers along with 5 μM ZOL by the transurethral and intravesical routes on Day 4 through 8 after cancer cell transplantation [46]. In our previous study, we used an in vivo orthotopic xenograft model to test a protocol based on weekly bladder instillation of γδ T cells, as this is a clinically acceptable schedule [47]. The results of these studies showed that intravesical administration of ex vivo-expanded γδ T cells combined with ZOL inhibits the growth of bladder cancers and prolongs survival significantly. Administration of ex vivo-expanded γδ T cells into a local cavity, rather than systemically, is one strategy that improves the antitumor effects of γδ T cells for clinical application.

## 4. Other Interactions between γδ T Cells and Cancer Cells

γδ T cells recognize not only PAgs via the γδ TCR, but also stress-associated antigens via the natural killer (NK) group 2 member D (NKG2D) receptor; as for natural killer cells, this method of recognition is MHC unrestricted [48,49,50,51,52,53]. In 1999, Bauer et al. reported that MHC class I chain-related molecule A (MICA) is a functional ligand that stimulates the NKG2D receptor [49]. In addition to MICA, the MICB and UL16-binding proteins 1–4 (ULBP 1–4) in human NKG2D ligands, as well as interactions between these ligands and the NKG2D receptor, are important for cancer cell recognition and γδ T cell-mediated cytotoxicity [51,52,53]. Anticancer agents inhibit immune function in cancer patients, mainly through bone marrow suppression [54]. However, recent studies show that some agents amplify the cytotoxic effects of immune cells against cancer cells [55]. Anticancer agents induce the DNA damage response, which in turn upregulates expression of NKG2D ligands [56]. Todaro et al. reported that low concentrations of anticancer agents 5-fluorouracyl and doxorubicin sensitize colon cancer-initiating stem cells to γδ T cell-mediated cytotoxicity via NKG2D receptor:ligand interactions [57]. Lamb et al. showed that temozolomide (TMZ), the main chemotherapeutic agent used to treat GBM, increases expression of NKG2D ligands on TMZ-resistant glioma cells, making them more susceptible to recognition and lysis by γδ T cells [58]. In our previous study, we showed that pretreatment of an orthotopic xenograft model with low-dose gemcitabine upregulates expression of MICA/B in bladder cancer cells and increases the cytotoxic effects of γδ T cells plus ZOL [47]. Molecularly targeted agents also could affect NKG2D ligands. Huang et al. reported that tyrosine kinase inhibitors, sorafenib and sunitinib, markedly increased NK cells cytotoxicity against multidrug-resistant nasopharyngeal carcinoma cells in association with up-regulation of NKG2D ligands, MICA, MICB, and ULBP1-3 [59]. Inhibition of epidermal growth factor receptor (EGFR) pathway also leads to induction of NKG2D ligands. Kim et al. reported that EGFR inhibitors, gefitinib and erlotinib enhanced the susceptibility to NK cell mediated lysis of lung cancer cells by induction of ULBP1 by inhibition of protein kinase C (PKC) pathway [60]. In the γδ T cells field, Story et al. reported that proteasome inhibitor bortezomib significantly increased expression of ULBP 2/5/6 in both acute myeloid leukemia (AML) and T-cell acute lymphoblastic leukemia (T-ALL) cells, and enhanced ex vivo expanded γδ T cell-mediated killing of these cells [61]. Histone deacetylase (HDAC) inhibitors, which are epigenetic agents, are also candidates for combined therapy with γδ T cells. Skov et al. reported that HDAC inhibitors upregulate NKG2D ligands on the surface of several cancer cells [62].

Expression of Fas ligand (FasL) and TNF-related apoptosis-inducing ligand (TRAIL) is upregulated in activated γδ T cells [63]. FasL interacts with CD95, also called Fas or APO-1, which was the first death receptor within the apoptotic chain to be molecularly characterized [64]. CD95 is expressed by various human cancer cells; ligation of CD95 by FasL activates the caspase cascade, which initiates cancer cell apoptosis. TRAIL interacts with five receptors (TRAIL-Rs): death receptor 4 (DR4), DR5, decoy receptor 1 (DcR1), DcR2, and osteoprotegerin [65,66,67,68,69]. Death receptors DR4 and DR5 contain a cytoplasmic region known as the death domain, which enables these receptors to initiate cytotoxic signals when engaged by TRAIL [70]. For these reasons, upregulation of CD95 or death receptors DR4 or DR5 in cancer cells might enhance γδ T cell-mediated cytotoxicity. Several anticancer agents upregulate CD95 or death receptors in cancer cells, thereby sensitizing cancer cells to apoptosis mediated by FasL and TRAIL. Shankar et al. report that paclitaxel, vincristine, vinblastine, camptothecin, etoposide, and doxorubicin upregulate DR4 and DR5 in prostate cancer cells, leading to augmentation of TRAIL-induced apoptosis via caspase activation [71]. Mattarollo et al. reported that etoposide, cisplatin, and doxorubicin upregulate CD95 and DR5 in various cancer cells, and that ex vivo-expanded NK cells kill sensitized targets via FasL- and TRAIL-mediated mechanisms [72]. Indeed, they showed that pretreatment of target cells with anticancer agents increased cytotoxicity to 60–70% (compared with the 5–30% observed when either chemotherapy or NK cells were used alone).

**Figure 2:**
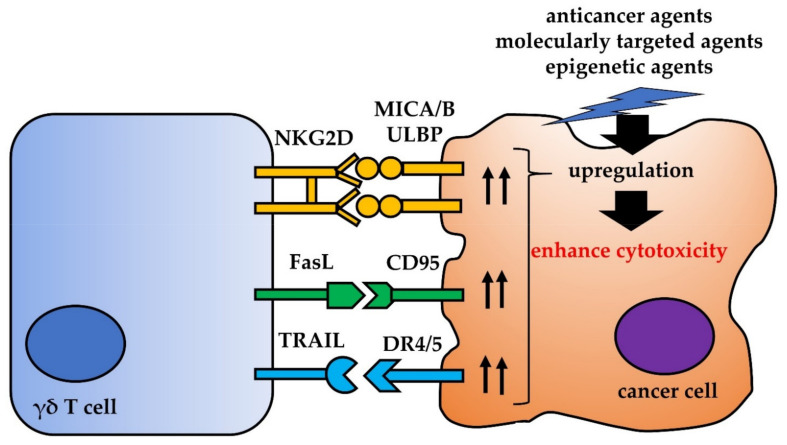
Interaction between γδ T cells and cancer cells. Anticancer agents, molecularly targeted agents, and epigenetic agents upregulate ligands that activate γδ T cells, thereby increasing cytotoxicity.

Thus, combination therapy with γδ T cells plus anticancer agents, molecularly targeted agents, and epigenetic agents are a promising strategy to improve the antitumor effects of γδ T cells for clinical application (Figure 2).

## 5. The Tumor Microenvironment (TME) Limits the Cytotoxicity of γδ T Cells by Promoting Their Regulatory Functions, by Secreting Immunosuppressive Cytokines, and by Inhibiting Immune Checkpoint Molecules

**Figure 3:**
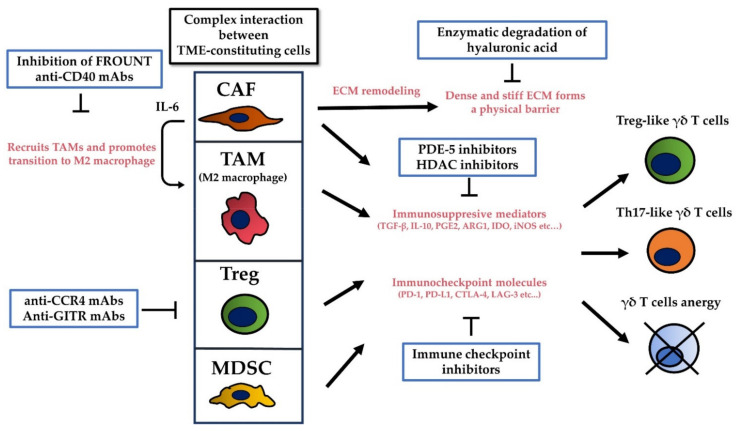
Cells in the TME induce polarization and anergy of γδ T cells in various ways (with red font) and some potential strategies to overcome negative effect from the TME are conceivable (in the blue boxes).

Several studies demonstrate the plasticity of γδ T cells. After activation by PAgs, γδ T cells promote a Th1 immune response by secreting TNF-α and IFN-γ; however, γδ T cells can be polarized into cells with properties similar to those of Th2 cells, Th17 cells, or regulatory T cells (Tregs) [73,74,75,76]. Unlike monolayer 2D models and mouse models injected with tumor cells, an actual tumor comprises not only cancer cells but also an extracellular matrix (ECM), stromal cells (such as fibroblasts and mesenchymal stromal cells), vascular networks, and immune cells such as T and B cells, NK cells, and tumor-associated macrophages (TAM). This is the TME. The TME plays a significant role in the subsequent evolution of malignancy [77]. For example, the TME harbors various cytokines, chemokines, growth factors, inflammatory mediators, and matrix remodeling enzymes to facilitate crosstalk between TME-constituting cells [78]; this environment can promote polarization of γδ T cells into Th17-or Treg-like cells that produce IL-17 and transforming growth factor (TGF)-β, which favor cancer cell proliferation [79,80]. IL-17-producing γδ T cells induce angiogenesis and support cancer progression [81,82]. TGF-β secreted by Treg cells can negatively regulate γδ T cells [83]. Moreover, the TME harbors various immunosuppressive cells (Figure 3).

Cancer-associated fibroblasts (CAFs), which are recruited to the tumor stroma by growth factors secreted by cancer cells, are key components that maintain an immunosuppressive TME. CAFs produce matrix-crosslinking enzymes and mediate ECM remodeling, resulting in a dense and stiff ECM [84]. The dense and stiff ECM compresses intratumoral blood and lymphatic vessels to increase interstitial tissue pressure, which induces hypoxia and impedes delivery of anticancer agents. The dense and stiff ECM also forms a physical barrier that prevents immune cells from infiltrating the cancer [85]. Provenzano et al. reported that hyaluronic acid (HA) is the primary determinant of the ECM barrier. They showed that enzymatic degradation of HA reduces interstitial tissue pressure to facilitate tumor penetration by gemcitabine, leading to improved antitumor effects in preclinical pancreatic ductal adenocarcinoma transgenic mouse models [86]. HA targeting might permit efficient delivery of γδ T cells to the tumor, thereby improving the E/T ratio on the tumor site. CAFs produce various immunosuppressive cytokines and factors such as IL-6, TGF-β, and prostaglandin E2 (PGE2) [87,88]. IL-6 recruits TAMs and promotes their transition to an immunosuppressive phenotype (i.e., M2 macrophages). CAFs can also inhibit activation of cytotoxic T cells and NK cells directly by expressing inhibitory immune checkpoint molecules such as programmed death-ligand (PD-L)1 and PDL-2 [89].

Myeloid-derived suppressor cells (MDSCs) also play a crucial role in maintaining an immunosuppressive TME. They are converted from immature myeloid cells in the bone marrow by inflammatory mediators released by cancer and immune cells and are recruited to the tumor site through interaction between C-C motif receptors (CCR) and their respective chemokines, such as C-C motif chemokine ligand. They produce different immunosuppressive mediators such as arginase-1 (ARG1), indoleamine 2,3 dioxygenase (IDO), and nitric oxide synthase (iNOS), all of which induce T cell anergy via different pathways [90]. Sacchi et al. reported that MDSCs inhibit IFN-γ production by PAgs-activated γδ T cells and suppress their cytotoxic activity [91]. Several strategies to target MDSCs have been investigated. Blocking migration of MDSCs is one strategy for targeting this cell type. CCR5 plays a key role in migration of MDSCs. The interaction between CCR5 and its ligand CCL5 supports tumor growth and invasion, and migration of MDSCs to the tumor site; tumor growth and invasiveness are suppressed by targeting the CCR5-CCL5 interaction [92,93,94]. Inhibiting MDSCs-producing immunosuppressive mediators is another strategy for targeting MDSCs. Serafini et al. reported that sildenafil and tadalafil, both of which are inhibitors of phosphodiesterase-5 (PDE-5), increase antitumor cytotoxic T lymphocyte activity and act synergistically with adoptive vaccine-primed CD8^+^ T cell therapy to delay tumor outgrowth in preclinical mouse models by downregulating ARG1 and iNOS activity [95]. Entinostat, a class I HDAC inhibitor, is another candidate agent that neutralizes MDSCs-producing immunosuppressive mediators. Orillion et al. reported that entinostat reduced the expression of ARG1, iNOS, and COX2 by MDSCs, and that the combination of entinostat plus anti-PD-1 antibodies increased survival and delayed tumor growth significantly in several preclinical mouse models [96]. Combination of γδ T cell immunotherapy with PDE-5 inhibitors and HDAC inhibitors is a good strategy for overcoming the immunosuppressive effects of MDSCs.

Tregs, which suppress aberrant immune responses against self-antigens, promote immune evasion of the TME. Infiltration of tumor tissue by a large number of Tregs is often associated with a poor prognosis. They not only exert immunosuppressive activity indirectly by releasing soluble inhibitory molecules such as TGF-β and IL-10, but also directly by inhibiting effector T cells via immune checkpoint receptor cytotoxic T lymphocyte antigen-4 (CTLA-4) and lymphocyte activation gene-3 (LAG-3) [97,98]. Molecules that are relatively specific for Tregs are good candidates for targeting Tregs in combination with γδ T cell immunotherapy. Several studies suggest that an anti-CTLA-4 monoclonal antibody (mAb) predominantly targets Treg cells and strengthens antitumor immune responses [99,100,101]. Moreover, the clinical efficacy of ipilimumab, a mAb specific for CTLA-4, correlates with a reduction in Treg numbers in tumor tissue [102,103]. CCR4 is expressed predominantly by effector Tregs, which are the most abundant cell type among FOXP3^+^ T cells in tumor tissue; in addition, CCR4 ligands produced by cancer cells or by infiltrating macrophages appear to be involved in migration and infiltration of Tregs into various tumor tissues [104,105]. Sugiyama et al. reported that anti-CCR4 mAb treatment selectively depleted effector Tregs and efficiently induced tumor antigen-specific CD4^+^ and CD8^+^ T cells both in vitro and in vivo [106]. Glucocorticoid-induced TNF receptor-related protein (GITR) is another molecule expressed by Tregs. Ko et al. reported that administration of an agonistic anti-GITR mAb affects tumor-infiltrating Tregs and evokes a potent antitumor immune response, which can eradicate established mouse tumors without eliciting overt autoimmune disease [107].

TAMs also play a pivotal role in the TME by behaving as M2 macrophages; these cells secrete anti-inflammatory factors such as IL-10, TGF-β, and vascular endothelial growth factor (VGEF)-A [108]. These inhibitory cytokines cause cancer cells to become refractory to immunotherapy. Therefore, therapeutic strategies to target TAMs might be effective. Inhibiting differentiation of systemic monocytes once they enter tumor tissue is one strategy to target TAMs. Interaction between CCR2 on monocytes with its ligand (CCL2) induces migration of monocytes from the circulation to the tumor tissue and promotes tumor proliferation. The cytoplasmic protein, FROUNT, binds directly to activated CCR2 and facilitates monocyte infiltration. Inhibition of FROUNT decreased the number of TAMs in an osteosarcoma mouse model [109,110]. Reprogramming of TAMs, i.e., transdifferentiating M2 macrophages to M1 macrophages, is an alternative strategy to target TAMs for cancer immunotherapy. First, M1 macrophages are induced by IFN-γ, and then combined treatment with IL-2 and anti-CD40 induces a switch from an M2 to an M1 phenotype [111]. Moreover, a recent study shows that PD-1 expressed by TAMs inhibits antitumor immunity [112]. Therefore, anti-PD/PD-L1 therapies are expected to have a direct effect on TAMs.

Among these TME-targeting therapies, therapeutic antibodies specific for inhibitory immune checkpoint molecules are an attractive strategy for overcoming the immunosuppressive effects of the TME; this is because various inhibitory immune checkpoint molecules are associated with immunosuppression by various TME-constituting cells. Therapeutic antibodies specific for PD-1, PD-L1, and CTLA-4, namely immune checkpoint inhibitors, have had a huge impact on cancer immunotherapy over the past decade [113,114,115,116]. The combination of adoptive γδ T cells plus immune checkpoint inhibitors is a hopeful strategy for improving their cytotoxicity because PAgs-stimulated γδ T cells express PD-1 [117] and Rossi et al. reported that blockade of PD-1 can boost antitumor effect of γδ T cells against follicular lymphoma [118]. However, we recently reported that PD-1 blockade did not increase the cytotoxicity of γδ T cell against PD-L1 ^high^ solid cancer cells and PD-L1 knockdown did not increase the cytotoxicity [119]. The augmentation effect of blockade of PD-1/PD-L1 axis is still controversial. Further studies should investigate how other inhibitory immune checkpoint molecules such as CTLA-4, IDO, and LAG-3, mediate their immunosuppressive effects against γδ T cells, and how these immunosuppressive effects can be circumvented.

## 6. Cancer Stem Cells (CSCs) Could Mediate Resistance to γδ T Cell Immunotherapy

According to the American Association for Cancer Research (AACR), CSCs are defined as cells within a tumor that possess the capacity to self-renew and to cause the heterogeneous lineages of cancer cells that comprises the tumor [120]. CSCs are a rare cell population within the tumor, but they are spared after conventional therapy because they are resistant and have the capacity to self-renew, ultimately causing tumor relapse and metastasis. Recent studies indicate that CSCs in various solid tumors play an important role in tumor resistance to conventional chemotherapy and radiotherapy [121,122,123]. Therefore, unsatisfactory clinical responses reported by past clinical trials of γδ T cell immunotherapy against various advanced and recurrent cancers might be due to the presence of CSCs. Moreover, CSCs can modulate immune cell activity by interacting with the TME. Jinushi reported that chemoresistant CSCs promote M2 macrophage differentiation through interferon-regulatory factor-5 (IRF5)- and macrophage-colony stimulating factor (M-CSF)-dependent mechanisms [124]. Schatton et al. reported that malignant melanoma CSCs possess the capacity to inhibit IL-2-dependent T cell activation and support induction of Tregs [125]. In addition, CSCs secrete several immunosuppressive cytokines into the TME, including TGF-β, IL-10, IL-4, and IL-13 [126,127]. CSCs also express high levels of immune checkpoint molecules, which enable them to evade to immune system [128]. Few studies have investigated the relationship between CSCs and γδ T cells. Previously, we generated prostate cancer spheres and used them to examine the cytotoxicity of *ex vivo*-expanded γδ T cells against sphere-derived prostate cancer cells. Sphere-derived prostate cancer cells were resistant to ex vivo-expanded γδ T cells; in addition, their stem cell markers, including CD133, NANOG, SOX2, and OCT4, were upregulating compared with those of parental cells [129]. These results suggest that ex vivo-expanded γδ T cells will not be effective against CSCs. Further research is needed to clarify the mechanisms underlying the resistance of CSCs to human γδ T cells.

## 7. Novel Forms of γδ T Cell Therapy Overcome Current Therapeutic Limitations

Recently, several strategies have been developed to improve the antitumor effect of γδ T cell immunotherapy. The use of a bispecific antibody, which is typically equipped with a first specificity for an antigen expressed by cancer cells and a second specificity for an activating molecule on effector cells [130], improved the cytotoxicity significantly. Hoh et al. reported that EpCAM/CD3 bispecific antibody enhanced γδ T cell -mediated lysis of hepatoblastoma and paediatric hepatocellular carcinoma cells in spheroid culture models [131]. Oberg et al. reported that ex-vivo expanded γδ T cell administration with the HER2/Vγ9 bispecific antibody significantly reduced the growth of pancreatic cancer and colon cancer in preclinical models [132,133]. They also reported that tribody [(HER2)_2_xCD16], which comprises two HER2-specific single chain fragment variables fused to a fragment antigen biding directed to the CD16 antigen expressed on γδ T cells and NK cells, enhanced γδ T cells and NK cells-mediated lysis of HER2-expressing tumor cells, such as pancreatic ductal adenocarcinoma, breast cancer, and autologous primary ovarian tumors [134]. Bispecific antibodies may be promising strategy to overcome current therapeutic limitations. Chimeric antigen receptor-transduced γδ T cells (CAR-γδ T cells) is another novel strategy to overcome current therapeutic limitations. Chimeric antigen receptors (CARs) are usually derived from single-chain variable fragments (scFvs) of antibodies specific for tumor antigens and transduced using viral vectors. Unlike TCRs, which have narrow range of affinities, CARs typically have a much higher and broader range of affinities [135], thus enabling the CAR-γδ T cells to recognize tumor epitopes independently on their TCR. Deniger et al. reported that polyclonal γδ T cells with CD19-specific CAR-γδ T cells enhanced killing of CD19^+^ tumor cells compared with CAR^neg^ γδ T cells in vitro, and CD19-specific CAR-γδ T cells reduced CD19^+^ leukemia xenografts in mice [136]. CAR-T cell immunotherapy has an off-target effect problem. Fisher et al. designed GD2-specific CAR-γδ T cells in order to limit the toxic effects on normal cells. GD2 is abundantly expressed on the surface of neuroblastoma cells and on several other cancer cell types. In this study, γδ T cells recognized the tumor antigen, then the monoclonal antibody against GD2 recognized GD2 and activated the downstream signal domain to exert antitumor effects. Consequently, GD2-expressing neuroblastoma cells which engaged γδ TCR were efficiently lysed, whereas cells that expressed GD2 equivalently bud did not engage γδ TCR were untouched [137]. Currently, several clinical studies have been ongoing (Table 2). CAR-γδ T cells are expected to be a new type of γδ T cell immunotherapy in the future.

## 8. Conclusions

In this review, we have discussed different ways of activating γδ T cells, along with various strategies aimed at improving their antitumor effects during clinical application. γδ T cell-based immunotherapy is very attractive because these cells show cytotoxic effects against various cancer types, both in vitro and in mouse models. However, clinical trials have reported limited clinical benefit. In vivo activation of γδ T cells by systemic administration of PAgs or N-bis, along with exogenous interleukin (IL)-2, is well tolerated; however, the clinical benefits appear to be mild to moderate, likely due to anergy and exhaustion of activation-induced γδ T cells. However, adoptive immunotherapy using ex vivo-expanded γδ T cells could be achieved by repeated administration of activated γδ T cells, although it is difficult to acquire adequate numbers of activated γδ T cells from some patients. Further research into the mechanisms underlying this problem is needed. Another problem with adoptive immunotherapy conferred by ex vivo-expanded γδ T cells is that systematic intravenous administration of these cells does not achieve a high E/T ratio at the target tumor site. Administration of ex vivo-expanded γδ T cells into a local cavity resolves this problem and is a promising approach to making the most out of their cytotoxic potential. Moreover, pretreatment with anticancer agents, molecularly targeted agents, and epigenetic agents sensitizes cancer cells to γδ T cells by upregulating expression of several stress-induced ligands. Immunosuppression of γδ T cells by the TME and CSCs is less clear-cut, and might operate via multiple mechanisms; however, they affect the immune system via common inhibitory immune checkpoint molecules. Therefore, co-immunotherapy with γδ T cells plus immune checkpoint inhibitors is one strategy that may improve cytotoxicity. Bispecific antibodies and CAR-γδ T cells are novel strategies which are expected to overcome current therapeutic limitations. Further basic studies of the immunosuppressive effects of the TME and CSCs on γδ T cells, along with clinical studies examining administration into local cavities, combination therapy with anticancer agents, molecularly targeted agents, epigenetic agents, and bispecific antibodies, and CAR-γδ T cell immunotherapy are needed to ensure successful clinical application of γδ T cell-based immunotherapy.

## Figures and Tables

**Table 1:** γδ T cell-based clinical trials.

Author	Year	Tumor	Interventions	Phase	Ref. or Clinical Trials. Gov Identifier
Wilhelm et al.	2003	MM, NHL	Pam + IL-2 (in vivo)	Pilot study	[27]
Kobayashi et al.	2006	RCC	Ex-vivo γδ T cell + IL-2	Pilot study	[33]
Kobayashi et al.	2007	RCC	Ex-vivo γδ T cell + ZOL + IL-2	I/II	[34]
Dieli et al.	2007	Prostate cancer	ZOL/ZOL + IL-2 (in vivo)	I	[26]
Bennouna et al.	2008	RCC	BrHPP + IL-2 (in vivo)	I	[31]
Abe et al.	2009	MM	Ex-vivo γδ T cell + ZOL + IL-2	Pilot study	[36]
Meraviglia et al.	2010	Breast cancer	ZOL + IL-2 (in vivo)	I	[29]
Bennouna et al.	2010	Solid cancer	BrHPP + IL-2 (in vivo)	I	[30]
Nakajima et al.	2010	NSCLC	Ex-vivo γδ T cell + ZOL + IL-2	I	[37]
Lang et al.	2011	RCC	ZOL + IL-2 (in vivo)	Pilot study	[28]
Nicol et al.	2011	Solid cancer	Ex-vivo γδ T cell + ZOL	I	[35]
Sakamoto et al.	2011	NSCLC	Ex-vivo γδ T cell + ZOL + IL-2	I	[39]
Noguchi et al.	2011	Solid cancer	Ex-vivo γδ T cell	Pilot study	[40]
Kanzmann et al.	2012	RCC, MM, AML	ZOL + IL-2 (in vivo)	I/II	[32]
Izumi et al.	2013	Colorectal cancer	Ex-vivo γδ T cell	Pilot study	[41]
Wada et al.	2014	Gastric cancer	Ex-vivo γδ T cell + ZOL (intraperitoneal injection)	Pilot study	[38]
Kakimi et al.	2014	NSCLC	Ex-vivo γδ T cell	I	[42]
Ghigo et al.	2020	Solid cancerHematopoietic/Lymphoid cancer	ICT01 (anti-BTN3A mAbs)/ICT01 plus pembrolizumab	I	NCT04243499

MM: multiple myeloma; NHL: non-Hodgkin’s lymphoma; RCC: renal cell carcinoma; NSCLC: non-small-cell lung cancer; AML: acute myeloid leukemia; Pam: pamidronate; IL-2: interleukin-2; ZOL: zoledronate acid; BrHPP: bromohydrin pyrophosphate; BTN3A: Butyrophilin subfamily 3 member A; mAbs: monoclonal antibodies.

**Table 2:** CAR-γδ T cell-based clinical trials.

Clinical Trials. Gov Identifier	Interventions	Cancers	Phase
NCT02656147	Anti-CD19-CAR-γδ T cell	Leukemia and lymphoma	I
NCT04107142	NKG2DL-targeting CAR-γδ T cell	Solid cancer	I
NCT04702841	CAR-γδ T cell	Relapsed and refractory CD7 positive T cell-derived malignant tumor	I
NCT04796441	CAR-γδ T cell	AML	Not Applicable

CAR: chimeric antigen receptor; NKG2DL: natural killer group 2 member D ligand; AML: acute myeloid leukemia.

## Data Availability

Not applicable.
